# *In-situ* one-step synthesis of carbon-encapsulated naked magnetic metal nanoparticles conducted without additional reductants and agents

**DOI:** 10.1038/srep38652

**Published:** 2016-12-06

**Authors:** Jun Kang, Yeonwon Kim, Hye-min Kim, Xiulan Hu, Nagahiro Saito, Jae-Hyuk Choi, Myeong-Hoon Lee

**Affiliations:** 1 Division of Marine Engineering, Korea Maritime and Ocean University, Busan 606–791, Korea; 2 Division of Marine Mechatronics, Mokpo National Maritime University, Mokpo 58628, Korea; 3 Graduate School of Engineering, Nagoya University, Nagoya 464–8603, Japan; 4 College of Materials Science and Engineering, Nanjing Tech University, Nanjing 211816, China

## Abstract

C-encapsulated highly pure Ni, Co, and Fe magnetic nanoparticles (MNPs/C) were synthesized by an innovative one-step *in-situ* plasma in liquid method (solution plasma processing, SPP) without any additional reductants, agents, or treatment. Successful encapsulation of MNPs was demonstrated by using inductively coupled plasma-atomic emission spectrometry and cyclic voltammetry techniques. The obtained X-ray diffraction patterns and transmission electron microscopy images corresponded to MNPs with average diameters of 5 nm and good crystalline structure. The C capsules with spherical shapes (containing onion-like layers) were characterized by uniform sizes (ranging from 20 nm to 30 nm) and chain-like morphologies. The synthesized MNPs/C exhibited superparamagnetic properties at room temperature and might be utilized in data storage, biomedical, and energy applications since various NPs (including bimetallic ones) could be easily prepared by changing working electrodes. This study shows the potential of SPP to be a candidate for the next-generation synthesis method of NPs/C.

Increasing interests have been devoted to nanoscale magnetic material because of large different properties compared with their bulk one, which is due to fundamental change in characteristics such as superparamagnetism, large surface-to-volume ratio, and high surface energy^1^. In particular, excellent magnetic materials such as iron-metal group (Ni, Co, Fe) have attracted much attention due to their unique properties^2^ such as high saturation magnetization with low coercivity, easy separation under external magnetic field^3^,^4^, as well as a wide range of promising applications such as data storage^5^, highly sensitive magnetic sensors^6^, and spintronic devices^7^. In addition, they are of great interest for biomedical uses including magnetic separation of hyperthermia during tumor therapy^8^, therapeutic drug delivery^9^, biological entities^10^, food analysis^11^, and contrast enhancement agents for magnetic resonance imaging^12^,^13^.

However, the utilization of bare MNPs is accompanied by serious problems resulting from the oxidation due to contact with air, water, and acids. Moreover, the high surface energy of NPs (caused by their large surface to volume ratio) facilitates particle agglomeration and makes the prevention of MNPs from physical and chemical degradation at conventional experimental conditions very difficult. Therefore, in order to increase particle stability and preserve large magnetic moment values, MNPs must be protected by an additional surface coating, which is stable at high temperatures and chemically inert in air, water, and acid environments.

For these reasons, the encapsulation strategy (which utilizes a core-shell structure) was studied to preserve the specific properties of MNPs and to overcome their above-mentioned limitations. To date, various shell materials made of polymers, silica, and C have been developed^14^,^15^. However, the core-shell nanostructures manufactured from silica and polymers are not perfect due to the (i) dissolution of silica coatings at strong basic conditions and (ii) low thermal stability of organic polymers. In contrast to these materials, C has many advantages, such as high stability under various physical and chemical conditions, good biocompatibility^16^,^17^,^18^, high electrical conductivity, and relatively low manufacturing costs. Moreover, C surface can be modified by various functional groups (including OH and COOH ones), which enhance its dispersibility in polar solutions^19^ and produce ternary hybrid structures^20^.

Various techniques for C encapsulation have been investigated including arc discharge method^21^, chemical vapor deposition^22^, cumulative method^23^, explosions^24^, microwave heating^25^, laser-assisted irradiation^26^, thermal plasma processing^27^, spray pyrolysis^28^, and hydrothermal method^29^. However, all of them require harsh growth conditions, multiple steps, sophisticated equipment, and high energy consumption. Furthermore, these techniques are limited in terms of scalability and economics because of the demanding synthetic conditions and generally low yields. The MNPs/C has been also produced by one step process in a liquid solvent^30^,^31^,^32^,^33^,^34^,^35^. However, these methods have disadvantages such as requiring a reducing or capping agent, sophisticated experimental apparatus and additional carbonization process as well as long synthesis time.

In order to overcome these problems, we proposed a simple synthesis method for MNPs containing C shells (MNPs/C), which is based on solution plasma processing (SPP). Recently, SPP has attracted much attention as a promising method for the synthesis of a large variety of nanomaterials (such as metal and metal oxide NPs^36^,^37^,^38^) and preparation of carbonaceous compounds^39^,^40^. The advantages of SPP (as compared with the above-mentioned methods) include a simple experimental setup, short time processing (in the range of several minutes), and standard operational conditions (room temperature and atmospheric pressure)^41^. However, the most important benefit of SPP is the extremely high purity of prepared materials, which can be achieved due to the absence of catalysts and additional agents.

The purpose of the present study is to simplify the currently used multiple-step processes by applying innovative SPP and to obtain MNPs/C with extremely high purity. We report the synthesis of Co, Ni, and Fe NPs encapsulated in onion-like C shells. The proposed method uses a bipolar power supply, pure benzene as C precursor, and a pair of magnetic metal electrodes. The structural characterization of the synthesized materials revealed that the obtained MNPs were encapsulated in onion-like C shells and exhibited a uniform size distribution, average diameters of less than 5 nm, and high crystallinity. In addition, the MNP structural properties and mechanism of the utilized synthetic process were discussed in detail.

## Results

### Synthesis of MNPs/C

In a previous work, we had successfully synthesized C-supported NPs by SPP via a one-step reaction^42^,^43^,^44^. In this study, we have prepared C-encapsulated NPs by varying major process variables, including frequency, voltage, and pulse width. Before the NP synthesis, an Au electrode was utilized to find the optimal NP encapsulation parameters since Au is characterized by very intense oxygen reduction reaction (ORR) and oxygen evolution reaction (OER) activities. Therefore, if the resulting Au NPs were successfully encapsulated in a C shell, they would not exhibit any ORR/OER peaks during CV testing. Thus, the best encapsulation parameters could be easily obtained just by comparing the corresponding CV curves.

**Figure 1:**
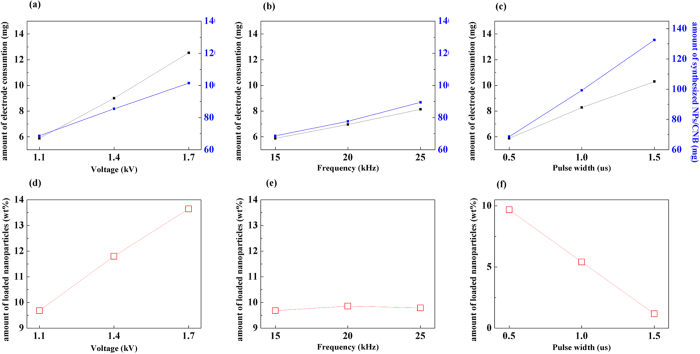
Dependences of the (**a**–**c**) electrode consumption and weights of the synthesized NPs/C and (**d**–**f**) fractions of the loaded NPs on various experimental parameters.

**Figure 2:**
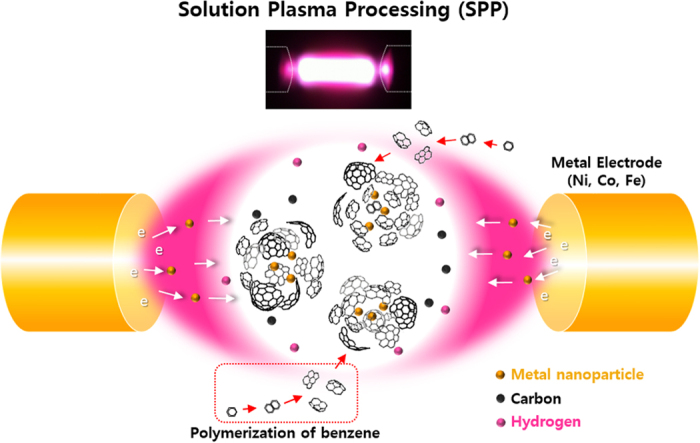
A mechanism of the NP/CNBs formation.

Figure 1 shows the effect of various experimental parameters on the composition of Au NPs/C. The amount of deposited Au NPs, which was estimated by inductively coupled plasma-atomic emission spectrometry (ICP−AES, Perkin-Elmer, PE OPTIMA−3300 DV) increased with increasing voltage and frequency (Fig. 1a and b) and decreased with an increase in the pulse width (Fig. 1c). In addition, the related amount of electrode consumption (corresponding to the weight of sputtered NPs, Fig. 1f) was lower than that of the synthesized NPs/C (in contrast to the voltage and frequency dependences; see Fig. 1d and e). The obtained results indicate that the C weight significantly exceeded that of sputtered NPs, and the synthesis rate of C flakes was more affected by the pulse width. Thus, it can be assumed that long plasma-on time per cycles activate C flake polymerization. If the pulse width is set to a high value, the produced NPs will most likely be surrounded by the multitude of C flakes, resulting in their encapsulation (the corresponding synthesis mechanism of MNPs is described in Fig. 2).

**Figure 3:**
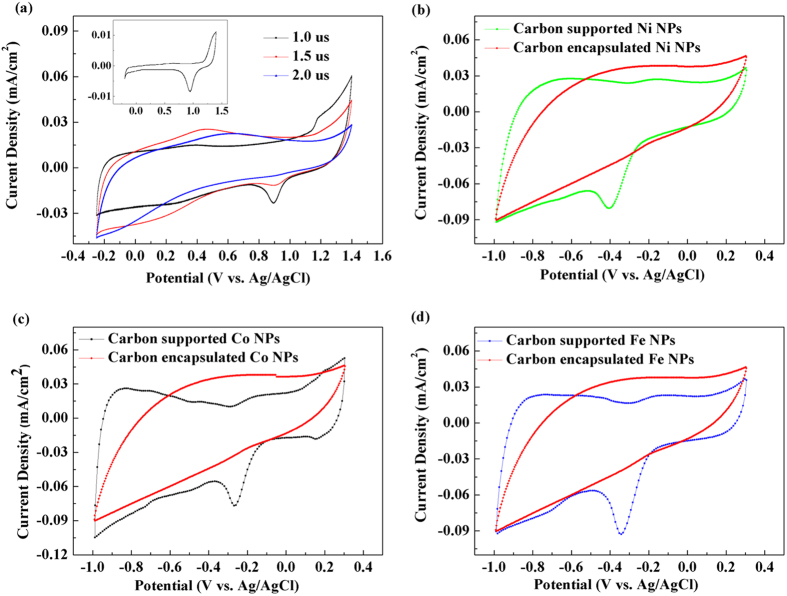
(**a**) CV curves for the Au NPs/C synthesized using the corresponding bulk metal electrodes at various pulse widths (the inset). (**b**–**d**) CV curves for the C-supported and encapsulated Ni, Co, and Fe NPs.

In order to confirm that the NPs synthesized at high values of the pulse width were not supported on C, a cyclic voltammetry (CV) method was applied to the Au NPs/C prepared at various pulse widths. Figure 3a displays the CV curves obtained for the Au NPs/C, which clearly show that the observed peaks gradually disappear with increasing pulse width, indicating the successful C encapsulation of NPs. Based on these results, MNPs/C were prepared at the same conditions (corresponding to the pulse width of 2.0 μs) and then compared with the C-supported MNPs samples synthesized at a low pulse width of 0.5 μs. Figure 3b–d show the CV curves obtained for these samples, indicating that the C-encapsulated MNPs do not exhibit any ORR peaks (in contrast to the supported MNPs).

**Figure 4:**
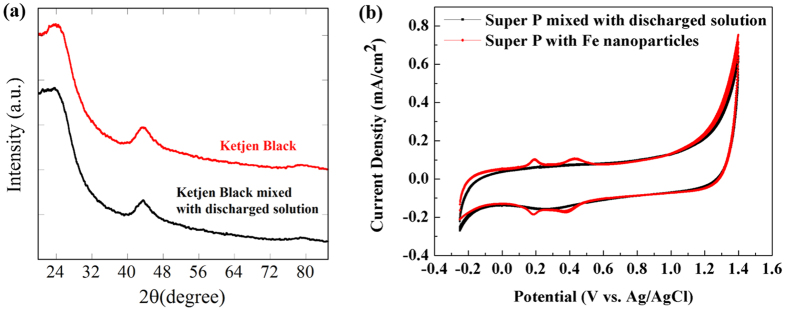
(**a**) XRD patterns obtained for the discharged solution with the Fe electrode. (**b**) CV curves for the discharged solution with the Fe electrode and Super P-supported Fe NPs.

In order to confirm that all the sputtered NPs were C-encapsulated, the discharged solution was filtered and mixed with other C materials (Ketjenblack EC600Jd), which did not support any metal NPs. After drying, X-ray diffraction (XRD) analysis was conducted. Figure 4a shows the XRD pattern obtained for the discharged solution with an Fe electrode, indicating that all the synthesized Fe NPs were successfully C-encapsulated (it did not contain any peaks related to the Fe NPs).

To support above results, additional experiments were conducted. We prepared Fe NPs dispersed solution by discharging Fe electrode in ethanol, and it was mixed with other C materials (Super P). The Super P was also mixed with the discharged solution. These two samples were characterized by cyclic voltammetry (CV) to compare the ORR/OER peaks by Fe NPs. Figure 4b shows the CV data and it shows that the discharged solution does not exhibit any ORR/OER peaks (in contrast to the Fe NPs/Super P). Therefore, it can be convinced that all the synthesized Fe NPs were successfully C-encapsulated. This means that there is no wasted NPs precursor (wire) during synthesis of NPs/C. This can be a major advantage for the synthesis of platinum metals.

### Morphology of MNPs/C

**Figure 5:**
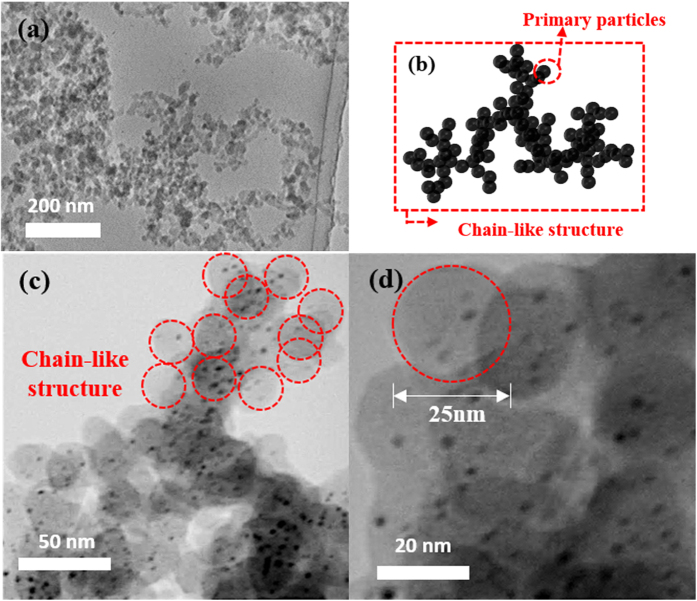
(**a**) TEM image and (**b**) BF−TEM images of the MNPs/C.

**Figure 6:**
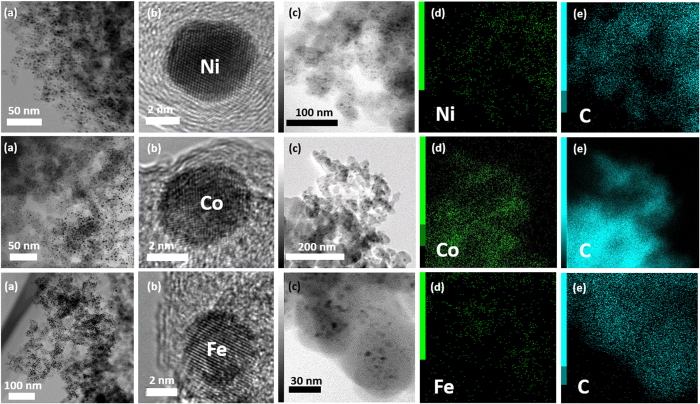
(**a**) BF−TEM, (**b**) HR−STEM, and (**c**–**e**) EDS mapping images of the MNPs/C.

**Figure 7:**
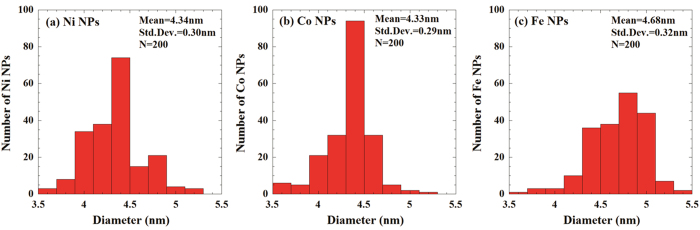
Size distribution of MNPs.

The morphology of the synthesized MNPs/C was investigated using the transmission electron microscopy (TEM; Fig. 5a), bright field scanning TEM (BF−STEM; Figs 5c,d and 6a), and HR−TEM images (Fig. 6b). The obtained TEM and BF-TEM images show that the observed C capsules exhibit spherical shapes with a chain-like morphology and uniform sizes ranging from 20 nm to 30 nm. In addition, BF−TEM images indicate that MNPs are remarkably uniform and well dispersed inside a C capsule. They are characterized by spherical shapes, narrow size distribution (Fig. 7), and average particle diameters of 4~5 nm.

On the other hand, the crystallites of carbon shell by SPP with low frequency is consisted of intermediate structures between graphite and amorphous state, also referred as turbostratic structure, which was different from the ABAB order of the bulk graphite crystal (Figure S1a)^39^. In addition, it is assumed that the carbon shells on NPs are formed by vigorous physical reactions by interactions between the sputtered NPs and the polymerized carbon flake. In other words, the polymerized carbon flake precipitated on nanoparticles with the rapid quenching by surrounded solution and thus some of carbon shells could not have enough time to undergo graphitation. Therefore, these carbon shells can be formed as amorphous-like turbostratic structure around the NPs as shown in Fig. 6b.

In addition, the recorded HR−TEM images clearly confirmed the existence of stable well-crystallized MNPs (without any aggregation), and the energy dispersive spectroscopy (EDS) mapping images (Fig. 6c–e) demonstrated their uniform distribution inside the C shells.

**Figure 8:**
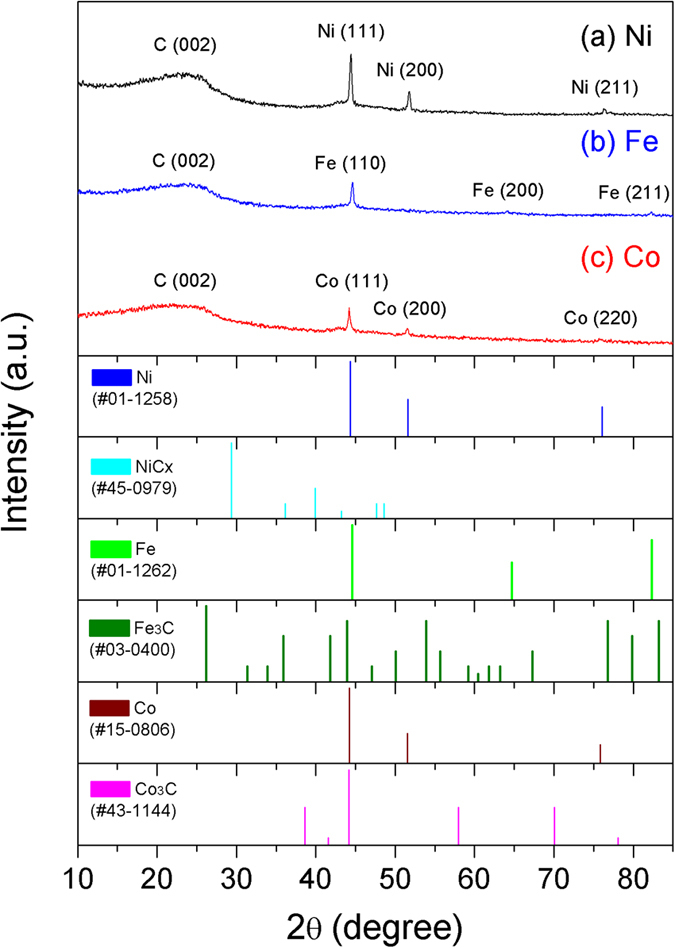
XRD patterns obtained for various MNPs/C.

The structural characterization of the SPP-synthesized MNPs/C was performed by XRD (the typical patterns are shown in Fig. 8). The diffraction pattern for Ni/C corresponded to the peaks observed at 2θ = 44.37°, 51.6°, and 76.08°, which matched the (1 1 1), (2 0 0), and (2 2 0) planes of fcc Ni (see ICDD file no. 45–0979). The Co/C reflections are represented by the peaks at 2θ = 44.22°, 51.52°, and 75.85°, attributed to the (1 1 1), (2 0 0), and (2 2 0) planes of fcc Co (ICDD file no. 15–0806). Both the obtained XRD patterns suggest that the Ni and Co NPs synthesized by SPP contained purely crystalline structures. For the Fe/C NPs, the obtained pattern was dominated by the intense peak at 2θ = 44.6°, which was attributed to the (1 1 0) plane of bcc Fe (ICDD file no. 01–1262). In addition, no major peaks corresponding to other chemical species were observed, indicating that the synthesized product contained pure Fe NPs without any oxides or carbide.

Using the broad peak areas obtained from the XRD patterns, average particle sizes were calculated using Scherrer’s formula^45^:


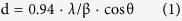


where D was the average particle size; 0.94 was the shape factor (Scherrer’s constant), which was generally used for cubic systems; λ was the wavelength of X-ray radiation (0.15406 nm), β was the full width at half maximum intensity measured in radians, and θ was Bragg’s angle. According to the data listed in Table 1, the synthesized MNPs were smaller than 10 nm, and their parameters were close to the values obtained from the TEM observations.

### Magnetic properties

**Figure 9:**
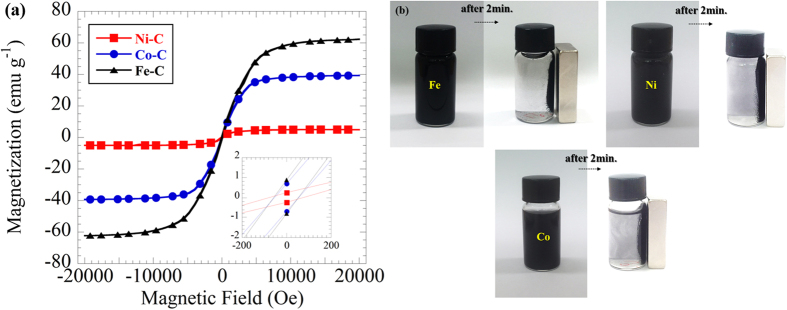
(**a**) Magnetization curves obtained for the SPP-synthesized Co−C, Ni−C, and Fe−C MNPs. (**b**) Photos of MNP/C in alcohol under the effect of a magnet bar outside the bottle, showing the MNPs/C are magnetically separable.

The magnetic properties of the SPP-synthesized MNPs/C studied at room temperature were characterized by the magnetization curves depicted in Fig. 9(a). The NPs, which were originally dispersed in an aqueous solution via shaking or ultrasonic vibration, were easily assembled by applying a magnet for 1 min. Once the magnetic domains of conventional ferromagnetic materials were aligned in the applied magnetic field, they started exhibiting magnetic memory and were no longer able to return to the initial state without applying external energy. However, no such hysteresis loops were observed for the resultant samples in the applied field, which were characterized by the negligible magnitudes of magnetic coercivity equal to 60 Oe (Co−C), 80 Oe (Ni−C), and 78 Oe (Fe−C), while their remanent magnetization values amounted to 0.67, 0.026, and 0.8 emu/g, respectively. The obtained data originated from carbon encapsulated magnetic nanoparticles under a diameter of 5 nm as mentioned in TEM observation, indicating the presence of single-domain particles and their superparamagnetic nature^46^,^47^,^48^. In order to demonstrate the magnetic performance of the MNPs/C in a liquid phase, a magnet bar was placed outside glass bottle. Figure 9(b) clearly show that the MNPs/C could move under the magnetic force and magnetically separable.

## Discussion

Highly pure MNPs were successfully encapsulated in onion-like C shells via the *in-situ* one-step SPP process. The ICP−AES, CV, and XRD studies revealed that all the synthesized MNPs were perfectly C-encapsulated, while the XRD TEM data showed that the average crystallite sizes of MNPs were around 5 nm. The performed magnetic measurements indicated the superparamagnetic properties of the MNP samples at room temperature. The obtained results confirmed that SPP was a very simple and facile method, which could be utilized for the large-scale synthesis of MNPs/C. It can also be applied to the C encapsulation of various types of NPs (including bimetallic ones) via changing the metal electrodes of an SPP system. Thus, SPP exhibits great potential as a candidate for the next-generation synthetic methods, and the prepared MNPs/C can be used in electromagnetic devices, cancer treatment, drug delivery, and magnetic resonance imaging applications.

## Methods

### Experimental setup and sample preparation

**Figure 10:**
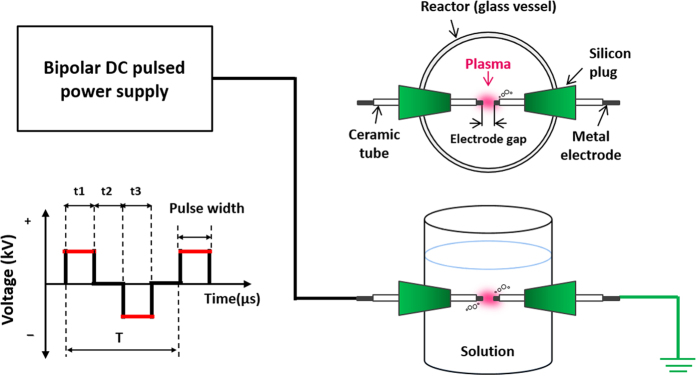
A schematic illustration of the SPP method.

All experiments were performed at atmospheric pressure and room temperature (Fig. 10 shows a schematic illustration of the SPP system used for the synthesis of MNPs/C). A pair Co, Ni, or Fe rods with diameters of 1 mm (purity 99.9%, Nilaco Corp., Japan) was used as electrodes to discharge plasma and MNPs precursor. In order to conserve energy, the electrodes were insulated by ceramic tubes with a protruded length of 1 mm measured from the tube tips. After that, they were placed in a glass vessel (a 100 mL beaker with a diameter of 5 cm and height of 7 cm) containing 70 mL of C benzene precursor (purity 99.5%, Kanto Chemical Co., Inc.), and the distance between the two electrode tips was set to 0.5 mm. Bipolar high-voltage pulses of 1.6 kV were applied to the electrodes by using a bipolar DC-pulsed power supply (Kurita, Japan), while the pulse width and frequency were fixed at 2.0 μs and 15 kHz, respectively.

### Characterization of MNPs/C

In order to identify whether the resulting NPs were encapsulated or C-supported, CV experiments were performed using an electrochemical analyzer (model HZ5000, Hokuto Denko Inc., Japan). The utilized three-electrode system consisted of an MNPs/C working electrode (deposited onto a glassy carbon (GC) rod with a diameter of 2 mm), an Ag/AgCl KCl-saturated reference electrode, and a counter electrode made of Pt wire. The working electrode was prepared by ultrasonicating the mixture containing 10 mg of the prepared sample, 1 mL of ethanol, and 100 μL of Nafion solution (Sigma-Aldrich, 5 wt.%) until a homogeneous suspension was formed. The obtained mixture (10 μL) was spread on the GC electrode and dried at room temperature. The CV measurements were conducted using 100 mL of a 1 M O_2_-saturated H_2_SO_4_ electrolyte in the voltage range of −0.25 V to 1.4 V (with respect to the Ag/AgCl reference electrode) at room temperature and a scan rate of 50 mV s^−1^.

TEM (JEOL, JEM2500SE) observations were conducted at an applied voltage of 200 kV to study the microstructure, shape, and size of the synthesized MNPs/C. TEM samples were prepared by dropping the MNPs/C suspension onto a copper grid coated with an ultrathin (about 6 nm in thickness) amorphous C layer with subsequent drying in air for 24 h. Particle sizes were evaluated by averaging the diameters of 100 particles observed in the TEM images. HR−TEM images were recorded close to the Scherzer defocus conditions at a lattice resolution of 0.14 nm. XRD patterns were obtained by using a Rigaku Smartlab (Rigaku, Japan) instrument with Cu Kα radiation (λ = 0.154 nm) operating at a voltage of 40 kV and current of 40 mA (1.6 kW) to examine the NP crystal structure.

The magnetic properties of MNPs/C were investigated at room temperature by using a vibrating sample magnetometer (Toei Industry Co. Ltd., Japan).

## Additional Information

**How to cite this article**: Kang, J. *et al. In-situ* one-step synthesis of carbon-encapsulated naked magnetic metal nanoparticles conducted without additional reductants and agents. *Sci. Rep.* **6**, 38652; doi: 10.1038/srep38652 (2016).

**Publisher's note:** Springer Nature remains neutral with regard to jurisdictional claims in published maps and institutional affiliations.

## Supplementary Material

Supplementary Figure 1

## Figures and Tables

**Table 1:** MNP particle sizes calculated from the XRD peaks depicted in Fig. 7.

2θ (degree)	Plane	Crystallite size (nm)	Average crystallite size (nm)
		Ni/C	
44.21	(1 1 1)	5.8	5.8
51.37	(2 0 0)	5.6	
76.29	(2 2 0)	6.0	
		**Co/C**	
44.43	(1 1 1)	7.05	
51.31	(2 0 0)	5.8	6.05
75.39	(2 2 0)	5.3	
		**Fe/C**	
44.48	(1 1 0)	5.1	5.1
