# Supplementary material for: In-situ one-step synthesis of carbon-encapsulated naked magnetic metal nanoparticles conducted without additional reductants and agents
Source: Sci Rep. 2016 Dec 6;6:38652. doi: 10.1038/srep38652 (PMC5138636; doi:10.1038/srep38652)
Supplement: Supplementary Figure 1 [file srep38652-s1.doc]

In-situ one-step synthesis of carbon-encapsulated naked magnetic metal nanoparticles conducted without additional reductants and agents

Jun Kang^a*^, Yeonwon Kim^b^, Hye-min Kim^c^, Xiulan Hu^d^, Nagahiro Satio^c^ , Jae-Hyuk Choi^a^ and Myeong-Hoon Lee^a^,

^a^Division of Marine Engineering, Korea Maritime and Ocean University, Busan 606−791, Korea

^b^Division of Marine Mechatronics, Mokpo National Maritime University, Mokpo 58628, Korea

^c^Graduate School of Engineering, Nagoya University, Nagoya 464−8603, Japan

^c^College of Materials Science and Engineering, Nanjing Tech University, Nanjing 211816, China

*Correspondence to [email : [leemh@kmou.ac.kr](mailto:leemh@kmou.ac.kr)]").


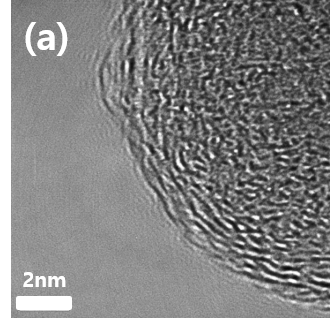

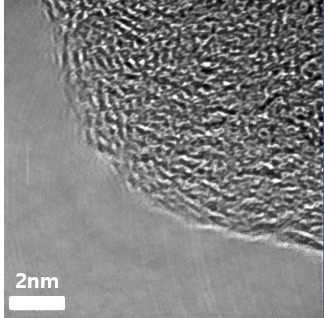

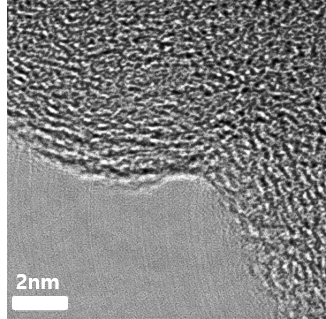


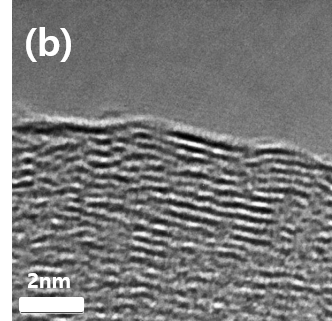

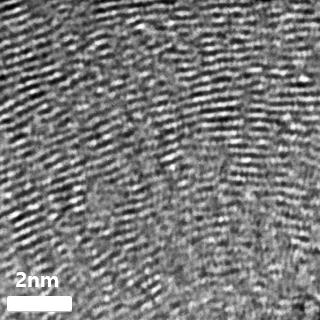

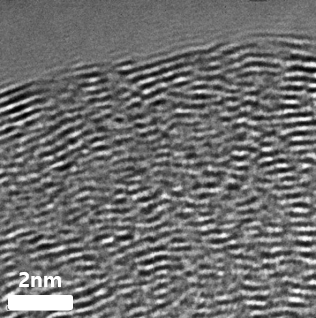


Figure S1. HR-TEM image of carbon shells synthesized with 25 kHz (a) synthesized with 65 kHz (b).
